# Chemotherapy in older patients with early breast cancer

**DOI:** 10.1016/j.breast.2024.103821

**Published:** 2024-10-11

**Authors:** Marcus Schmidt, Sibylle Loibl

**Affiliations:** a Department of Obstetrics and Gynecology, University Medical Center of the Johannes Gutenberg-University, Mainz, Germany; b GBG Forschungs GmbH, Neu-Isenburg, Centre for Haematology and Oncology, Bethanien, Frankfurt, Germany

**Keywords:** Early breast cancer, Older patients, Geriatric assessment, Chemotherapy, Estrogen receptor

## Abstract

The incidence of breast cancer increases with age. Particularly in ageing societies, breast cancer has a significant impact on both the older patient and the healthcare system. In older patients with early breast cancer, there is a complex interplay between (i) tumor biology, (ii) risk of recurrence, (iii) comorbidities, (iv) frailty, (v) life expectancy and (vi) patient expectations and preferences. Our treatment guidelines are often based on large meta-analyses that have shown that (neo)adjuvant chemotherapy improves the survival rate in early breast cancer in general. This is particularly important in triple-negative and HER2-positive breast cancer, but hormone receptor (HR)-positive, HER2-negative patients with a higher risk of recurrence also benefit from chemotherapy. However, most studies included younger and carefully selected patients. Since there is a positive correlation between age and estrogen receptor status, as well as between age and the number of concomitant diseases and the tolerability of chemotherapy, it is of great importance to evaluate the effects of additional (neo)adjuvant chemotherapy, especially in older patients with early-stage breast cancer. There are only a few studies in which only older patients with early breast cancer were included. On the whole, they show that older patients with HR-positive, HER2-negative tumors hardly benefit from chemotherapy in addition to endocrine therapy. In these patients, additional chemotherapy should be considered critically when weighing up the potential benefits and harms. However, this critical evaluation should not be confused with abandoning standard chemotherapy when it is feasible and clinically indicated based on geriatric assessment, risk assessment, and patient preference. The aim of our narrative review is to provide a concise overview of the evidence on chemotherapy in older women with breast cancer and place it in the context of geriatric assessment and risk evaluation in older HR-positive, HER2-negative patients with early breast cancer. This in turn should help to critically weigh up the risks and benefits of chemotherapy for the individual older patient with early-stage breast cancer, which should ultimately lead to more individualized and at the same time more evidence-based treatment recommendations that take into account the complex interplay of different and sometimes contradictory patient- and tumor-specific factors.

## Highlights

• Increasing incidence of older patients with early breast cancer.
• Incorporation of geriatric assessment important.
• Complex interaction of patient- and tumor-specific factors.
• Positive correlation between a patient's age and the gene expression of the estrogen receptor (ER).
• Reduced effect of chemotherapy in older patients with positive ER.

## 1 Introduction

From a global perspective, breast cancer is the most commonly diagnosed cancer (11.7 % of all cases) and the fifth most common cause of death from cancer (6.9 %) [1]. Age-specific incidence rates of breast cancer are rising, particularly in high-income countries [2].

**Fig. 1:**
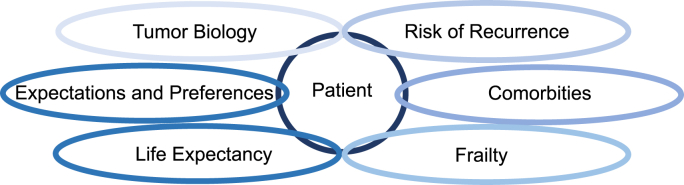
Complex interplay of tumor- and patient-related factors.

Importantly, the biology of breast cancer changes with increasing age. There is a well-documented association between increasing age at diagnosis and the presence of more favorable biological features of the tumor [[3], [4], [5], [6], [7]]. Especially, the gene expression of estrogen receptor-α (*ESR1*) shows a strong positive correlation with the age of breast cancer patients at diagnosis [8,9]. Patients with estrogen receptor(ER)-positive breast cancer are candidates for adjuvant endocrine treatment (ET) [10,11]. In principle, a patient's risk of recurrence can be further reduced by additional chemotherapy. Large meta-analyses on the effect of adjuvant chemotherapy (CT) in early breast cancer show that the relative risk reduction by chemotherapy is independent of classical clinicopathological factors such as estrogen receptor, tumor size, nodal status, degree of histological differentiation and age [12,13]. However, the absolute risk reduction depends on the baseline risk of recurrence and is influenced by competing mortality risks due to comorbidities. This is of course important for all breast cancer patients, but it is of the utmost importance for older patients, as comorbidities increase significantly with age [14]. In addition, chemotherapy has been shown to significantly accelerate the loss of physical function in older patients with early-stage breast cancer compared to breast cancer patients without chemotherapy or healthy controls [15]. Indeed, choice of therapy in older patients requires not only an assessment of the risk of the tumor but also of the patient's frailty and vulnerability. In addition, expectations of older patients often differ from those of younger patients (e.g. maintaining control, independence, quality of life) - " … to stay considered as a human being with a meaningful life" [16]. This makes it clear that chemotherapy poses a particular challenge for older patients with early breast cancer. This is due to a complex interplay between (i) tumor biology, (ii) risk of recurrence, (iii) comorbidities, (iv) frailty, (v) life expectancy and (vi) patient expectations and preferences (Fig. 1).

### 1.1 Geriatric assessment in older patients with breast cancer

A short geriatric screening such as G-8 with 8 items can help to identify patients who require a more detailed comprehensive geriatric assessment (CGA) (Table 1) [17]. The total G-8 score ranges from 0 (severely impaired) to 17 (not impaired at all). A threshold of 14 (versus >14) provides good sensitivity estimates for the G-8 of at least 80 % compared to reference tests, without degrading specificity below 60 %. Therefore, a G-8 score ≤14 should prompt a detailed CGA [17]. Indeed, as suggested by the European Society of Breast Cancer Specialists (EUSOMA) and the International Society of Geriatric Oncology (SIOG), a screening tool is recommended before making a decision about cancer treatment in older patients [18].

**Table 1:** The G-8 questionnaire.

Items	Score
A Has food intake declined over the past 3 months due to loss of appetite, digestive problems, chewing, or swallowing difficulties?	0 = severe decrease in food intake
	1 = moderate decrease in food intake
	2 = no decrease in foodintake
B Weight loss during the last 3 months	0 = weight loss >3 kg
	1 = does not know
	2 = weight loss between 1 and 3 kg
	3 = no weight loss
C Mobility?	0 = bed or chair bound
	1 = able to get out of bed/chair but does not go out
	2 = goes out
E Neuropsychological problems?	0 = severe dementia or depression
	1 = mild dementia
	2 = no psychological problems
F BMI? (weight in kg)/(height in m^2^)	0 = BMI <19
	1 = BMI 19 to <21
	2 = BMI 21 to <23
	3 = BMI ≥23
H Takes more than three prescription drugs per day?	0 = yes
	1 = no
P In comparison with other people of the same age, how does the patient consider his/her health status?	0.0 = not as good
	0.5 = does not know
	1.0 = as good
	2.0 = better
Age	0: >85
	1: 80–85
	2: <80

Abbreviations: BMI, body mass index.

The American Society of Clinical Oncology (ASCO) recently updated its recommendations for older patients, stating that a geriatric assessment (GA) should be performed on all cancer patients over the age of 65 to identify vulnerabilities or impairments [19]. 10.13039/501100023321GA includes physical and cognitive function, emotional health, comorbidities, polypharmacy, nutrition and social support. The Practical Geriatric Assessment (PGA) tool was developed to address the barriers to the routine use of GA in clinical practice https://old-prod.asco.org/sites/new-www.asco.org/files/content-files/practice-patients/. The PGA questionnaire has 23 items, 19 of which (e.g., "Does your health limit you in walking one block?") can be completed by the patient, 4 (i.e., nutrition, gait speed, mini-cog, chemo-toxicity) must be completed by the health-care provider.

The assessment of chemo-toxicity is particularly important for older breast cancer patients. Magnuson and colleagues developed and validated a risk tool to predict severe chemotherapy-related toxicities in 473 older (≥65 years) patients with early breast cancer who received chemotherapy [20]. 46 % developed grade 3–5 chemotherapy toxicities. They identified 8 independent weighted predictors, such as planned use of anthracyclines, stage II or III, planned treatment duration >3 months, abnormal liver function, low hemoglobin, falls, limited walking ability, and lack of social support (Table 2).

**Table 2:** Cancer and Aging Research Group-Breast Cancer (CARG-BC) score.

Risk predicor	Score
Planned anthracycline use	1
Stage II or III	3
Planned treatment duration >3 months	4
Abnormal liver function	3
Low hemoglobin	3
Falls	4
Limited walking	3
Lack of social support	3

The total Cancer and Aging Research Group-Breast Cancer (CARG-BC) risk score is the sum of each point(s) derived from eight independent clinical and geriatric predictors of grade 3–5 chemotherapy toxicity in older patients with early breast cancer. Each patient's total CARG-BC score can then be classified into three risk groups: low (0–5 points), intermediate (6–11 points), or high (≥12 points). The CARG-BC score reliably predicted grade 3–5 chemotherapy toxicity in older patients with early-stage breast cancer and was significantly associated with hospitalization, dose reduction, dose delays, early treatment discontinuation, and reduced dose intensity (P < 0.001).

A proven method to reduce chemotherapy-related toxicities is dose reduction and delay, which can eventually lead to low dose intensity. Sedrak and co-authors investigated the effects of dose intensity on survival in 322 patients aged ≥65 years with early breast cancer in the prospective HOPE study [21]. A low relative dose intensity (RDI) was defined as ≤ 85 %. It is well known that it is necessary to administer combination chemotherapy at a full dose (>85 %) to achieve clinical benefit [[22], [23], [24]]. RDI was defined as the ratio between the actual chemotherapy dose received and the standard dose intensity. The authors found that 21 % of patients had a low RDI, which was related to age ≥76 years, lower performance status, and anthracycline-based or cyclophosphamide, methotexate, fluorouracil (CMF) chemotherapy in multivariable Cox regression. Of note, variables such as prior radiation, primary granulocyte colony-stimulating factor (G-CSF) prophylaxis, or hormone receptor (HR) status were not associated with low RDI. A low RDI was significantly associated with poorer 5-year overall survival (80 % vs. 91 %; P = 0.02).

### 1.2 Randomized evidence for chemotherapy in older patients with early breast cancer

In the latest meta-analysis on the effects of chemotherapy in early breast cancer, the authors make it clear that "only a few patients over the age of 65 were included" [13]. Due to this uncertainty in the aggregated evidence in older breast cancer patients, it makes sense to focus on studies that investigate the effect of chemotherapy exclusively in patients over 65 years of age. Despite this obvious need, however, there are only very few studies investigating the effects of adjuvant chemotherapy exclusively in older patients with early breast cancer, and even fewer studies comparing chemotherapy versus no chemotherapy (Table 3).

**Table 3:** Randomized adjuvant chemotherapy trials in older patients with early breast cancer.

Reference	Treatment	N	Inclusion	ER/PR+	DFS HR (95 % CI)	OS
CALGB 49907 [25,26]	CMF or AC vs. Capecitabine	633	Age ≥65 Stage I-III	67 %	RFS 56 % vs. 50 % HR 0.80 (0.62–0.98) ER/PR + HR 0.66 (0.46–0.95) ER/PR- HR 0.89 (0.68–1.18)	62 % vs. 56 % HR 0.84 (0.66–1.07)
CASA [27]	PLD vs. LDMCM or NIL	77	Age ≥66 Stage I-III	0	78 % vs. 78 % 3-year BCFI	
ELDA [28]	Docetaxel vs. CMF	299	Age 65-79 Stage I-III	78 %	65 % vs. 69 % HR 1.21 (0.83–1.76)	HR 1.34 (0.80–2.22)
ICE II [29]	CMF or AC vs. nab-paclitaxel + capecitabine	391	Age ≥65 Stage I-III CCI ≤2	65.5 %	0.91 (0.49–1.71)	1.18 (0.52–2.66)
ASTER 70s [30]	CT + ET vs. ET	1089	Age ≥70 Stage I-III GGI high	100 %		90.5 % vs. 89.7 % HR 0.85 (0.64–1.13)
ICE [31]	Capecitabine + ibandronate vs. ibandronate	1409	Age ≥65 Stage I-III	81 %	78,8 % vs. 75 % HR 0.96 (0.78–1.19)	90.1 % vs. 87.6 % HR 0.87 (0.65–1.18)

Abbreviations: AC, doxorubicin/cyclophosphamide; BCFI, breast cancer-free interval; CCI, Charlson comorbidity indexCI, confidence interval; CMF, cyclophosphamide/methotrexate/5-flourouracil; CT, chemotherapy; DFS, disease-free survival; EC, epirubicin/cyclophosphamide; ER, estrogen receptor; ET, endocrine therapy; GGI, genomic grade index; HR, hazard ratio; LDMCM, low-dose metronomic cyclophosphamide + methotrexateOS, overall survival; PLD, pegylated liposomal doxorubicin; PR, progesterone receptor; RFS, relapse-free survival.

The first randomized phase-III trial in which only older patients with early breast cancer were treated was CALGB 49907 [25]. Patients were randomly assigned to standard chemotherapy or capecitabine. Most had an excellent performance status. For patients with hormone receptor-positive tumors, endocrine therapy was recommended after chemotherapy. Recruitment was terminated prematurely as an interim analysis predicted that capecitabine was inferior to standard chemotherapy. Patients randomized to capecitabine were twice as likely to relapse and almost twice as likely to die as patients randomized to standard chemotherapy (P = 0.02). Moderate to severe toxicities were more likely in patients randomized to standard chemotherapy (64 % vs. 33 %). After 10 years of follow-up, relapse-free survival (RFS) was 56 % and 50 %, respectively (hazard ratio [HR] 0.80; P = 0.03), and overall survival (OS) was 62 % and 56 %, respectively (HR 0.84; P = 0.16). Standard chemotherapy was superior to capecitabine in hormone receptor-negative patients (HR, 0.66; P = 0.02), but not in hormone receptor-positive patients (HR, 0.89; P = 0.43) [26]. The authors concluded that that standard chemotherapy with CMF or AC resulted in superior RFS compared with capecitabine in older women with early-stage breast cancer and that the major benefit of standard chemotherapy was seen among patients with hormone receptor-negative disease.

In the CASA study, Crivellari et al. randomized older patients with endocrine non-responsive early-stage breast cancer not suitable for a standard regimen between pegylated liposomal doxorubin (PLD) vs. low-dose metronomic cyclophosphamide + methotrexate (CASA-CM, n = 72) or PLD vs. no adjuvant chemotherapy (CASA-nil, n = 5) [27]. The study was prematurely terminated due to slow recruitment. Grade 3 adverse events occurred in 51 % of PLD patients and 34 % of patients on low-dose metronomic cyclophosphamide + methotrexate. The 3-year survival rate without breast cancer was 78 % in both study arms. Patients who received PLD had a poorer quality of life as well as poorer cognitive and physical performance than patients who did not receive PLD therapy. However, the authors concluded that both regimens may be a reasonable option for older and vulnerable patients with endocrine non-responsive tumors.

In an attempt to increase the efficacy of adjuvant therapy with taxanes, Perrone and colleagues randomized 299 older patients (65–79 years) at moderate to high risk of early breast cancer to standard chemotherapy with CMF or weekly docetaxel [28]. A geriatric assessment (GA) was performed and quality of life (QoL) was evaluated. Disease-free survival (DFS) (HR 1.21; P = 0.32) and OS (HR 1.34; P = 0.32) were similar in both groups. Importantly, there was no interaction between the treatment arms and the geriatric parameters. 70 % of patients treated with CMF had severe hematologic toxicities compared to 9 % with docetaxel (P < 0.0001). Conversely, non-hematologic toxicities such as diarrhea, allergies, fatigue or neuropathy were more frequently reported by patients treated with docetaxel (28 % vs. 19 %; P = 0.07). Overall quality of life was similar in both treatment groups, although docetaxel led to a significant worsening in various subscales for side effects of systemic therapy (e.g., future outlook, nausea and vomiting, diarrhea, loss of appetite, hair loss impairment and body image). The authors concluded that weekly docetaxel was not superior to CMF, worsened several areas of quality of life, and led to more non-hematologic side effects.

To further escalate adjuvant chemotherapy, the ICE-II trial randomly assigned non-frail patients aged 65 years and older with early-stage, intermediate-to high-risk breast cancer to either standard chemotherapy (CMF or EC) or weekly treatment with nab-paclitaxel + capecitabine [29]. 391 patients were treated. Adverse events of grade ≥3 occurred more frequently in patients treated with standard chemotherapy (90.9 %) than in those treated with nab-paclitaxel plus capecitabine (64.8 %) (P < 0.001). Especially, hematologic toxicities occurred more frequently with standard chemotherapy (88.4 % vs. 22.3 %; P < 0.001). Conversely, nonhematologic toxicities (e.g. hand-foot syndrome, diarrhea, mucositis and sensory neuropathy) occurred more frequently with nab-paclitaxel + capecitabine (58.5 % vs. 18.7 %; P < 0.001). Treatment discontinuation was observed in 6.6 % with standard chemotherapy versus 35.8 %. None of the geriatric parameters (i.e. Charlson Comorbidity Index [CCI], Vulnerable Elders Survey [VES-13], Instrumental Activities of Daily Living [IADL] and G-8) independently predicted moderate to severe toxicities or treatment discontinuations.

The phase-III ICE (ibandronate-capecitabine-elderly) study, in which bisphosphonates were obligatory, included patients aged 65 years or older with node-positive/high-risk node-negative early breast cancer [31]. 1409 patients were randomized to receive either capecitabine plus ibandronate or ibandronate alone for 2 years. Endocrine therapy was recommended for hormone receptor-positive patients. At a median follow-up of 61.3 months, the 5-year iDFS was 78.8 % for capecitabine + ibandronate versus 75.0 % for ibandronate alone (HR 0.96; P = 0.80). The OS was comparable in both study arms (HR 0.87; P 0.49). The effects were independent of age, nodal status, HR-status and geriatric assessments at baseline such as CCI or the VES 13 score. The addition of capecitabine caused significantly higher skin (14.6 % vs. 0.6 %; P < 0.01) and gastrointestinal toxicity (6.7 % vs. 1 %; P < 0.001). In summary, patients treated with bisphosphonates and, if HR-positive, with endocrine therapy had favorable survival. However, a significant effect of capecitabine was not observed. Although this large study only included chemotherapy in one arm, the results are affected by the significantly lower efficacy of capecitabine compared to standard chemotherapy shown in CALGB 49907.

These randomized trials involved older breast cancer patients who had a higher risk of recurrence based on conventional clinicopathological risk assessment. Genomic testing is now increasingly being used to determine whether patients with ER-positive, HER2-negative cancer require adjuvant chemotherapy in addition to adjuvant ET [10]. The not yet fully published Unicancer ASTER 70s trial randomized older (≥70 years) estrogen receptor (ER)-positive, HER2-negative, early-stage breast cancer patients with a high genomic grade index (GGI) between CT + ET and ET alone [30]. 1969 patients were included and 1099 with high GGI were randomized. After a median follow-up of 5.8 years, the 4-year OS was 90.5 % in the CT + ET group and 89.7 % in the ET alone group (HR 0.85; p = 0.2538). A potential limitation of ASTER 70s is that 20.5 % of patients randomized to chemoendocrine therapy did not receive chemotherapy. In the per-protocol analysis, the 4-year OS was 91 % in the CT + ET group and 89.3 % in the ET-alone group (HR 0.73; p = 0.03). In summary, in the intent-to-treat analysis there was no statistically significant benefit for overall survival after surgery in ER-positive, HER2-negative breast cancer with high GGI when CT was performed in addition to ET. However, the results of the per-protocol analysis do not rule out a potential benefit of chemoendocrine therapy in older patients with early high-risk breast cancer.

Meanwhile, studies have shown that adding CDK4/6 inhibitors to ET improves survival not only in advanced breast cancer but also in early breast cancer [32,33]. Adjuvant Palbociclib in Elderly Patients With Breast Cancer (Appalaches) is a phase-II study to determine the efficacy of the combination of at least 5 years of endocrine therapy and 2 years of palbociclib as adjuvant treatment in place of adjuvant chemotherapy followed by endocrine therapy in older (≥70 years) patients with ER-positive, HER2-negative early breast cancer stages II-III [34]. The addition of a CDK4/6 inhibitor is an obvious and promising treatment for older patients with early breast cancer, but the results of the Appalachies study are still pending.

Overall, these trials, which have some caveats in terms of design, treatment selection and risk assessment, suggest that older patients with HR-positive, HER2-negative early breast cancer may have limited benefit from chemoendocrine therapy. However, this should not be confused with forgoing standard chemotherapy when it is clinically indicated.

### 1.3 Risk assessment in older ER-positive, HER2-negative patients with early breast cancer

Risk assessment is crucial in assessing whether a patient benefits from chemotherapy. A commonly used online tool is PREDICT (https://breast.predict.nhs.uk/tool) which accurately predicts overall survival after surgery in early breast cancer [35]. Focusing on older breast cancer patients, the PREDICT model accurately predicted 5-year overall survival but overestimated 10-year predicted overall survival [36]. Particularly in older patients, the benefit of adjuvant chemotherapy depends not only on the traditional prognostic factors that characterize the tumor, but also on the risk of early mortality or toxicity and the general state of health of the patient in question.

To address this, a systematic review in older breast cancer patients included 173 studies, most of which looked at survival, toxicity and, to a lesser extent, patient-reported outcomes (PROMs) [37]. The authors found that various geriatric parameters such as gait speed, self-reported balance or walking difficulties, previous falls, and ADL (activity of daily life) dependency can be used to identify patients at risk of early mortality or treatment induced toxicity.

Based on these findings, the research group developed and validated a prognostic tool for 5-year recurrence, all-cause mortality and mortality from other causes in older patients with early invasive breast cancer. In addition, this instrument estimates the individual expected benefit of adjuvant systemic treatment [38]. The development cohort comprised 2744 patients with a mean age of 74.8 years and the validation cohort 13,631 patients with a mean age of 76 years. In addition to the clinicopathologic risk factors used in the well-known, comorbidities and geriatric parameters such as walking difficulties, dementia or cognitive impairment, polypharmacy and sensory deficits were included in the calculation of the final PORTRET tool. The PORTRET tool enabled accurate prediction of 5-year overall survival and recurrence in older patients with breast cancer. In addition, the expected benefit of adjuvant systemic treatments and the impact of comorbidities as a competing risk for other causes of mortality were estimated, supporting shared decision-making. Indeed, integration of information regarding the general health status in multigene prognostic models is crucial in older patients [18].

Gene expression profiling might be used for clinical decision making in patients with early breast cancer. Two large randomized trials investigated the benefit of adding chemotherapy to endocrine therapy in node-negative and node-positive patients with early HR-positive, HER2-negative breast cancer and a midrange 21-gene recurrence score [3,39]. Both studies showed that chemotherapy did not improve survival in postmenopausal patients, although very few of them were 70 years or older. A recent review including altogether 15 studies in patients aged 65 or older clinical confirmed the prognostic performance of Oncotype DX® and Prosigna® risk of recurrence in older patients with HR-positive, HER2-negative breast cancer [40]. Oncotype DX® was predictive for older patients, and chemotherapy could be spared in both node-positive and node-negative patients with early breast cancer. However, the authors also concluded that additional validation is needed before routinely using gene expression signatures for adjuvant chemotherapy decision making in older patients.

In a systematic review by the International Society of Geriatric Oncology on adjuvant chemotherapy and gene expression profiling (GEP) in older patients with (HR)-positive, HER2-negative breast cancer, the authors identified 8 publications on the use of chemotherapy and 5 publications on GEP [41]. The authors concluded that in older patients with HR-positive, HER2-negative early breast cancer, the survival benefit of adjuvant chemotherapy is unclear and GEP is used less frequently. Indeed, a recent recommendation stated that the evidence for multi-gene expression testing in older patients is insufficient and recommended the inclusion of information on general health status in older patients with early breast cancer [18].

## 2 Conclusion

Treatment decisions for older patients with early-stage breast cancer are complex, as the risk factors of the tumor must be weighed against the overall health of the older patient. It is generally recognized that chemotherapy has an important place in HR-negative, especially triple-negative, and HER2-positive breast cancer. Clearly, a geriatric assessment is advisable before chemotherapy is proposed for these molecular subtypes in older patients. Modifications (e.g. shorter duration of chemotherapy such as 4 cycles of anthracycline or docetaxel plus cyclophosphamide or 12 cycles of weekly paclitaxel plus trastuzumab in HER2-positive breast cancer, no dose-dense or dose-intensified chemotherapy) of regimens used in older patients compared to standard chemotherapy in younger patients are also advisable [18].

However, as mentioned above, the vast majority of older patients with early-stage breast cancer have HR-positive, HER2-negative tumors that are treated with endocrine therapy. In these patients, additional chemotherapy should be considered critically when weighing the potential benefits and harms However, this critical evaluation should not be confused with abandoning standard chemotherapy when it is feasible and clinically indicated based on geriatric assessment, risk assessment, and patient preference.

## CRediT authorship contribution statement

**Marcus Schmidt:** Writing – original draft, Investigation, Data curation, Conceptualization. **Sibylle Loibl:** Writing – review & editing, Investigation, Conceptualization.

## Ethical approval

An approval was not required for this review of previously published studies.

## Funding

No external funding has been received for the preparation of this review.

## Declaration of competing interest

MS reports grants and personal fees from Pierre-Fabre, grants, personal fees and non-financial support from Roche, grants, personal fees and non-financial support from Pfizer, grants and personal fees from Novartis, grants and personal fees from Astra-Zeneca, grants and personal fees from Eisai, personal fees from Amgen, grants, personal fees and non-financial support from Pantarhei Bioscience, grants, personal fees and non-financial support from BioNTech, grants from Genentech, personal fees from SeaGen, personal fees from Lilly, personal fees from Gilead, personal fees from Daiichi Sankyo, personal fees from Menarini Stemline, outside the submitted work; In addition, Dr. Schmidt has a patent EP: 2951317 Method for predicting the benefit from inclusion a taxane in a chemotherapy regimen in patients with breast cancer issued, and a patent EP2390370 A method for predicting the response of a tumor in a patient suffering from or at risk of developing recurrent gynecologic cancer towards a chemotherapeutic agent issued.

SL reports employment as Chief Executive Officer (CEO) at German Breast Group (GBG) Forschungs GmbH; institutional fees for advisory board membership for AbbVie, Amgen, AstraZeneca, Bristol Myers Squibb (BMS), Celgene, DSI, EirGenix, Gilead, GSK, Lilly, Merck, Novartis, Olema, Pfizer, Pierre Fabre, Relay Therapeutics, Roche, Sanofi and Seagen; institutional fees as an invited speaker for AstraZeneca, DSI, Gilead, Medscape, Novartis, Pfizer, Roche, Seagen and Stemline-Menarini; institutional research grants from AstraZeneca, Celgene, Daiichi Sankyo, Immunomedics/Gilead, Novartis, Pfizer and Roche; institutional funding from AbbVie, Greenwich Life Sciences and Molecular Health; institutional licensing fees from VMscope GmbH; a role as a steering committee member (non-financial interest) for AstraZeneca, Daiichi Sankyo, Immunomedics/Gilead, Novartis, Pfizer, Roche and SeaGen; a role as a principal investigator (PI) for Pfizer (non-financial interest); a non-remunerated advisory role for Arbeitsgemeinschaft Gynäkologische Onkologie (AGO) Kommission Mamma; a non-remunerated role as PI for PI Aphinity; non-remunerated membership of Die AGO, the American Society of Clinical Oncology (ASCO), German Cancer Society (DKG), ESMO and the ESMO Guidelines Committee; non-remunerated role as Chair of ESMO Breast; institutional patents for EP19808852.8, EP14153692.0, EP21152186.9 and EP15702464.7 (non-financial interest).
